# Lifestyle medicine: A positive approach to stemming the tide of non-communicable diseases in South Africa

**DOI:** 10.4102/safp.v63i1.5394

**Published:** 2021-10-26

**Authors:** Dave R. Glass

**Affiliations:** 1 Private Obstetrics/Gynaecology Practitioner, Port Shepstone, South Africa

**Keywords:** lifestyle medicine, disease reversal, medical education, non-communicable diseases, lifestyle changes, diet, exercise

## Abstract

Non-communicable diseases account for most deaths globally and they are the main reason for patient consultations. Conventional medicine focuses on pharmaceutical and surgical interventions to manage these diseases. Most management protocols recognise the foundational role of lifestyle changes, but practical lifestyle medicine interventions need to become part of the medical curriculum and part of each consultation to make an impact. This article presents the rationale for the launch of the South African Lifestyle Medicine Association as an important facilitator of that process.

## Introduction

According to a World Health Organization fact sheet dated 1st June 2018,^1^ non-communicable diseases (NCDs) kill 41 million people each year (71% of all deaths globally). The four main groups of such diseases are cardiovascular disease, cancer, respiratory disease, and diabetes. ‘NCDs are a result of a combination of various genetic, environmental and especially lifestyle factors, including smoking, alcohol abuse, unhealthy diets and physical inactivity’.^2^ The South African Non-communicable Diseases Alliance states:

NCDs, currently pose one of the biggest threats to health and development globally, particularly in low- and middle-income countries. It is predicted that unless proven interventions are rapidly implemented in countries … health care costs will increase exponentially, and severe negative consequences will ensue not only to individuals and families but to whole societies and economies. NCDs are already a major burden in South Africa (SA), but without added rigorous and timely action the health and development consequences may well become catastrophic. Immediate and additional, high quality, evidence based and focussed interventions are needed to promote health, prevent disease and provide more effective and equitable care and treatment for people living with NCDs at all levels of the health system.^3^

These are the sobering opening statements in the National Department of Health’s NCDs National Strategic Plan draft proposal released for comment on 18 May 2020. We propose that Lifestyle Medicine (LM) is one such ‘evidence-based and focused’ intervention that can respond to this threat.

The rate of increase in diabetes in Africa is one of the highest in the world^4,5^ and as of 2017, diabetes was the leading cause of death in females in SA.^6^ Heart disease has similarly high rates in SA compared to most Western countries (a mortality rate of 1:4).^7^ The incidence of obesity with its attendant risk of diabetes, hypertension (HTN) and atherosclerosis in SA is rising faster than in the West.^8^ Smoking incidences in SA have come down in some quarters but are static in others.^9^ Alcohol abuse is still a major problem in terms of loss of productivity, injuries, crime, and violent deaths.^10^ The incidence of cancer is rising, particularly the so-called cancers of lifestyle such as colon, breast, prostate, pancreas, and lung cancer.^11^

Already in 2013, efforts at changing the trajectory of NCDs in SA had begun through the Strategic Plan for the Prevention and Control of Non-Communicable Diseases 2013–2017. The 72-page document notes that the NCD epidemic can be prevented by reducing the underlying risk factors and implementing early detection and timely treatment. Three main components are outlined to achieve this goal: healthy lifestyle promotion, strengthening of health systems, and effective monitoring of patients and risk factors.^3^ These are also the components of LM.

Although public health interventions have made some gains, for example in reducing the number of smokers, thus decreasing the incidence of lung cancer and the number of young people who commence smoking, the implementation of other measures continues to be far too slow to impact the future health demands of our country. Bradshaw et al, speak of the race against time.^12^ An already constrained public health system is not able to afford the costs in mounting public health interventions, and the public is losing faith in promised improvements.^13^ Funding priorities are focused on infectious diseases (HIV and AIDS and tuberculosis), particularly international donor funding.^14^ The NCD epidemic in SA is an even greater burden because it is occurring concurrently with an ageing HIV-positive population. By 2030, NCDs will account for five times as many deaths as communicable diseases in low- and middle-income countries.^15,16^

## Challenges of tackling non-communicable diseases in South Africa

It is essential that tackling NCDs should be through both population-based strategies and individualised care. It is the responsibility of governments to provide policies, regulations and infrastructure to implement a healthy environment, and to restrict the availability and marketing of harm-producing substances, for example, tobacco, excess sugar intake, alcohol availability to minors, and so on.

The challenge comes with individualising health interventions to prevent and reverse lifestyle diseases. It is well known that knowledge, although important, cannot necessarily alter behaviour.^17^ For years, cigarette packages have announced the dire consequences of smoking, but it is doubtful that this has had a great deterrent effect, especially on seasoned smokers.^18^ Non-communicable diseases are best managed on an individual basis where they are holistically addressed, often with a team approach.^19^ This is exactly the strength of LM through coaching and facilitating self-determination.

## Evidence for lifestyle factors underlying non-communicable diseases

What are the origins of lifestyle interventions in the fight against NCDs? Although not the first mention of lifestyle intervention, arguably South Africa’s most prolific research scientist, Alex (ARP) Walker, together with Denis Burkitt and others, identified fibre deficiency as a major contributor to many of the common western NCDs already in the 1960s and early 1970s.^20^ Thus the foundations were laid in our understanding of the role that energy-dense processed foods and the greater consumption of animal products have played in the rapid increase of NCDs.^21^

But it was researchers such as Dean Ornish^22^ and Caldwell Esselstyn^23^ who were instrumental in proving that lifestyle interventions could not only arrest the progress of atherosclerosis, but also reverse the narrowing of coronary arteries. Roy Taylor, through the Counterpoint study^24^ published in 2011 demonstrated the rapid reversibility of Type 2 diabetes mellitus with a very low-calorie diet. Abundant other LM research has shown the dramatic benefits in terms of preventing, stabilising and in many cases reversing HTN, hyperlipidaemia, auto-immune disease, renal failure, non-alcoholic fatty liver disease, and even certain cancers. A comprehensive review of the effectiveness of LM is presented by Balazs Bodai et al.^25^

With this surge in research demonstrating the possibility of the reversal of hitherto relentlessly progressive NCDs, new hope was generated in the practice of medicine. The American College of Lifestyle Medicine was founded in 2004, followed by many sister organisations around the world, the latest being the South African Lifestyle Medicine Association (SALMA) in South Africa.

## Underlying principles of lifestyle medicine

Because lifestyle related diseases are caused by harmful lifestyle practices, the most rational approach is to address these factors through evidence-based lifestyle therapeutic approaches. The human organism has an extraordinary capacity to heal and regenerate through adopting the following principles: a predominantly whole food, plant-based diet, regular physical activity, adequate sleep, management of stress, avoidance of risky substance use, and use of other non-drug modalities that promote health and prevent disease. Lifestyle medicine recognises the role of conventional therapy, but secondary to addressing the underlying causes of disease.

Most guidelines for managing chronic diseases in SA mention the importance of LM. The SA HTN guidelines state: ‘All patients with HTN should receive lifestyle counselling … and this is the cornerstone of management’.^26^ Similar recommendations are found in the South African guidelines for type 2 diabetes and dyslipidaemia.

## Training of health care practitioners in lifestyle medicine

Current medical education in most countries of the world is deficient in addressing practically this ‘cornerstone of management’. Polak, et al., have written a comprehensive review on LM education.^27^ This education can take place in undergraduate training, but also needs to be made available to practising health practitioners through distance teaching courses, or continuing medical education (CME) interventions. It is important that practitioners (and this includes allied professionals) are taught the following skills:

- Perform comprehensive lifestyle assessments including risk factors.
- Assess patients’ readiness to change modifiable risk factors.
- Understand behaviour change theories and how to apply these in consultations.
- Have a working knowledge of the role of diet in the generation of lifestyle diseases and the pros and cons of various dietary approaches to healing.
- Understand the role of physical activity in promoting health and reversing chronic diseases, and understand the basics of exercise prescriptions.
- Understand sleep physiology and its role in health and disease prevention.
- Understand the role of stress and the principles of stress management.
- Have practical training in managing addictive behaviour.
- Understand the role of other non-drug modalities such as positive psychology, meditation and spiritual practices, mindfulness, social connectedness and living with purpose in health promotion.
- Understand the role of teamwork and group consultations in dealing with lifestyle diseases.

## Launch of South African Lifestyle Medicine Association

As part of a world-wide movement, SALMA has been established by a group of enthusiastic International Board of Lifestyle Medicine Certified Diplomates, and other passionate medical practitioners who have caught the vision. Lifestyle Medicine offers new hope and a sense of fulfilment in the practice of medicine, one that emphasises healing rather than just managing disease. The SALMA would like to spearhead a more comprehensive approach to the practice of medicine that fundamentally addresses the causes of NCDs in individual patients rather than just the complications.

The SALMA has been established with the following objectives in mind:

- Make LM training resources available for students in medicine and allied professions.
- Provide a forum for the sharing of experience and practical interventions.
- Promote evidence-based LM as foundational to healthcare vs. disease care, both amongst qualified practitioners as well as students.
- Advocate for the inclusion of LM in the medical curriculum in SA.
- Provide guidance/assist special interest groups to reach consensus on lifestyle guidelines for the management of chronic diseases.

To this end, SALMA would like to build a database of LM-oriented practitioners and organise future conferences and interactions to enhance communication and learning.

## Conclusion

No better concluding remarks could be made than by Sagner and fellow LM leaders:

In the modern era of evidence-based medicine, it is substandard practice not to offer the treatment option, or primary recommended therapy, of effective lifestyle medicine services. The provision of evidence-based, safer, more effective and less expensive lifestyle medicine services are the privilege and duty of every practitioner, patient and healthcare provider on every continent today. It will require the collective efforts of all parties to deliver patients, providers, and economies from the tyranny of the present high-cost, low-effectiveness system of lifestyle symptom care. To deliver effective care in today’s world requires treating the lifestyle causes as the foundation of care.^28^
